# A new mouse model highlights the need for better JAK inhibitors in myeloproliferative neoplasms

**DOI:** 10.1002/hem3.66

**Published:** 2024-04-02

**Authors:** Charles E. de Bock

**Affiliations:** ^1^ Children's Cancer Institute, Lowy Cancer Research Centre UNSW Sydney Kensington New South Wales Australia; ^2^ School of Clinical Medicine UNSW Sydney Kensington New South Wales Australia

The discovery that the gain of function *JAK2* ^*V617F*^ mutation is present in myeloproliferative neoplasms (MPNs) has led to numerous clinical trials assessing the efficacy of JAK inhibitors. Most notably, ruxolitinib, a combined JAK1/2 selective inhibitor, has gained approval in patients with myeolofibrosis (MF), and additional JAK2 inhibitors including fedratinib, pacritinib, and momelotinib also under evaluation for patients with MF. However, while these inhibitors demonstrate some clinical benefit, they do not adequately reduce the mutant clone fraction.^1^, ^2^ Consequently, a critical question for the field has been whether the lack of a durable response is attributed to either (i) the inability of current JAK inhibitors to completely block the pathway or (ii) the possibility that mutant clones are not entirely dependent on this activated pathway.

To address these two possibilities, a new study from the laboratory of Ross Levine, published in *Cancer Discovery*,^3^ developed an innovative mouse model of *Jak2* ^*V617F*^ alone or in combination with *Tet2* loss. The novel aspect of this mouse model lies in the ability to control the expression and genetic deletion of *Jak2* ^*V617F*^ allele from mutant clones upon development of MPN. To do so, it utilizes two orthogonal site‐specific recombinases which exert precise control over the temporal expression and deletion of the *Jak2* ^*V617F*^ allele.

The mouse model uses the well‐established Cre recombinase that recognises short nucleotide target sequences called Lox sites, in conjunction with the relatively new Dre recombinase which recognizes short nucleotide sequences called Rox sites. Importantly, the strategic arrangement and orientation of these sequences can lead to either flipping or deletion of the intervening DNA sequence. In this context, Dre recombinase is employed to initiate the expression of the *Jak2* ^*V617F*^ allele. Subsequently, a modified CreER recombinase, translocated to the nucleus upon tamoxifen treatment, can delete the *Jak2* ^*V617F*^ allele (Figure 1A). This intricate mouse model provided a powerful tool for comparing the durability of response between JAK inhibitors and the genetic loss of *Jak2* ^*V617F*^ in the context of MPNs.

**Figure 1:**
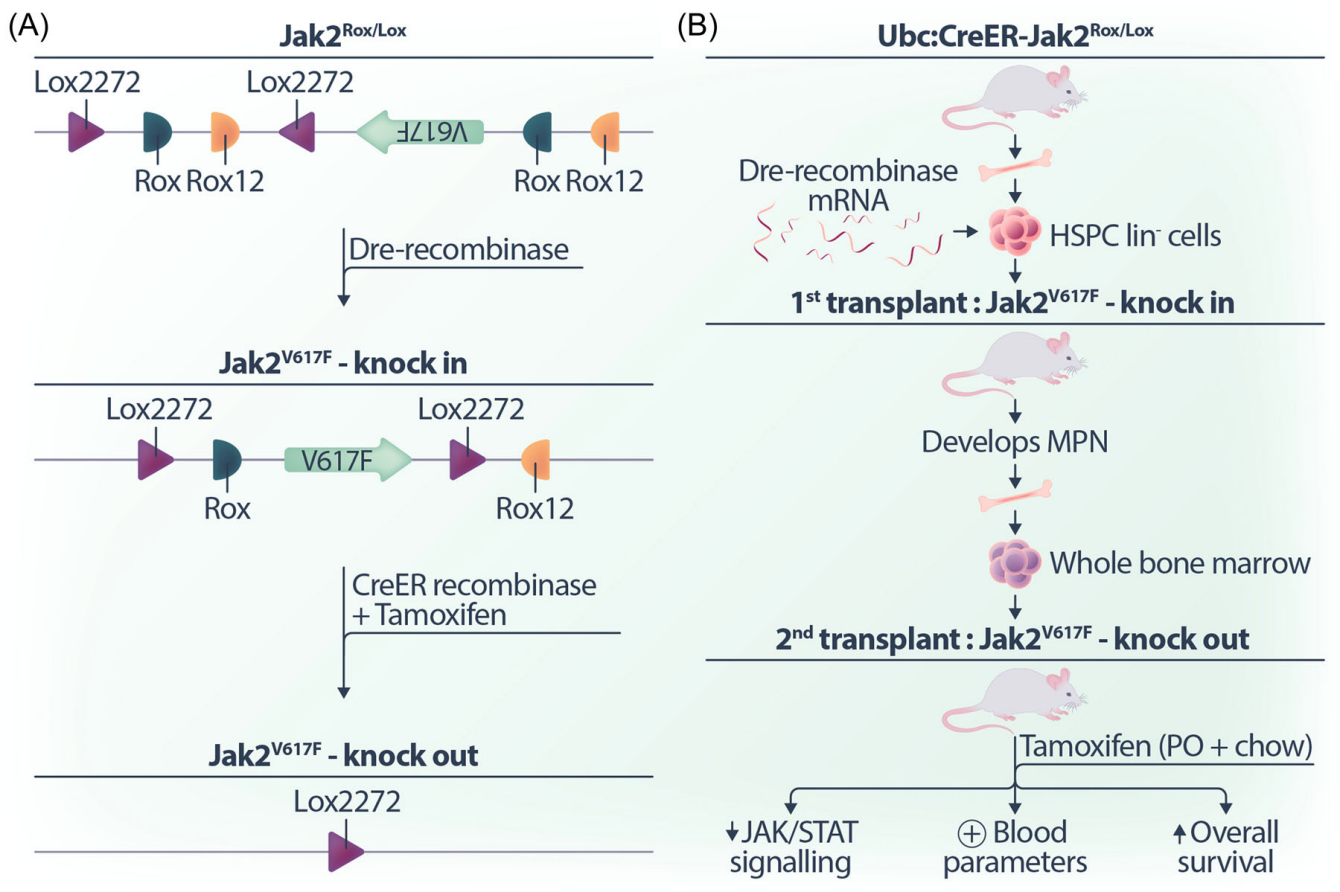
Inducible expression and deletion of *Jak2* ^*V617F*^ allele in a novel mouse model. (A) Schematic overview: A segment of the Jak2 locus is depicted, where the *Jak2* ^*V617F*^ allele is initially cloned in the antisense direction. The allele can be “flipped” to the sense direction via Dre recombinase expression, recognizing the Rox sequence motifs in opposing orientations. The subsequent deletion of the *Jak2* ^*V617F*^ allele occurs upon the introduction of Cre recombinase, recognizing the Lox sequence motifs in the same direction. (B) Model validation: Recipient mice transplanted with lineage‐negative hematopoietic stem and progenitor cells expressing the *Jak2* ^*V617F*^ allele exhibit a myeloproliferative neoplasm (MPN) phenotype. Upon transplanting the bone marrow of affected mice into secondary recipients and treating with tamoxifen to delete the mutant *Jak2* ^*V617F*^ allele, there is a reduction in JAK/STAT signalling, resolution of the hematopoietic system, and increased overall mouse survival.

To understand the role of Jak2^V617F^ in the initiation and maintenance in MPNs in vivo, lineage‐negative hematopoietic stem and progenitor cells were harvested from donor *Ubc:CreER‐Jak2* ^*Rox/Lox*^ mice. These cells were then electroporated with Dre recombinase‐encoding messenger RNA to induce Jak2^V617F^ expression and then transplanted into lethally irradiated recipient mice, that all went on to develop MPNs, confirming that Jak2^V617F^ is both sufficient and necessary for MPN development.

To investigate whether Jak2^V617F^ remained essential after the development of MPNs, the whole bone marrow of mice exhibiting MPN was injected into secondary recipients. At 12 weeks posttransplantation, the secondary recipient mice were treated with tamoxifen for Cre recombinase‐mediated deletion of the mutant *Jak2* ^*V617F*^ allele. The authors found that the genetic deletion of *Jak2* ^*V617F*^ not only reduced JAK/STAT signalling but also normalized key blood parameters and increased the overall survival of mice (Figure 1B).

Significantly, when extending the mouse model to include coincident loss of *Tet2^−/−^*, the genetic loss of *Jak2* ^*V617F*^ resolved hematological parameters to the same extent as observed in the single *Jak2* ^*V617F*^ mutant setting, thereby providing evidence that the dependence on Jak2^V617F^ persists even in the presence of other high‐risk mutations.

Having established that Jak2^V617F^ remains essential, the authors proceeded to compare the transcriptional and phenotypic effects of Jak2^V617F^ genetic deletion with the impact of ruxolitinib treatment. Their findings showed that ruxolitinib was unable to reduce JAK/STAT or inflammatory signalling to the same degree as genetic deletion. Additionally, with ruxolitinib treatment, hematological parameters did not show comparable improvement, nor was there any reduction in mutant lineage‐Sca1+cKit+ cell fraction. Taken together, this mouse model supports the hypothesis that the elimination of mutant clones requires JAK inhibitors with enhanced potency.

One approach to enhancing the potency of JAK inhibitors has been the clinical development of type II JAK2 inhibitors that block JAK2 in the “inactive form.” In the current study, the authors show that the type II JAK2 inhibitor CHZ868 exhibits significantly greater efficacy in improving hematological parameters compared to ruxolitinb. In an independent study, another strategy has been to repurpose ruxolitinib and a related JAK inhibitor baricitinib as cereblon‐directed JAK proteolysis‐targeting chimeras (PROTACs). Notably, these PROTACs demonstrated efficacy within a pre‐B‐ALL mouse model.^4^

Nevertheless, even with improved potency, several questions remain. These include whether (i) the level of tyrosine kinase inhibition can ever fully phenocopy complete genetic deletion; (ii) resistance mechanisms, such as acquired point mutations, are inevitable; and (iii) potent inhibitors might drive the outgrowth of clones with other high‐risk mutations excluding JAK2^V617F^? Interestingly, in relation to the third question, a recent study found that patients with clones containing high‐risk mutations in genes such as *TET2*, *DNMT3A*, and *ASXL1* but lacking JAK2^V617F^ did not impact prognosis.^5^

Overall, this new study provides a strong rationale for the ongoing development of more potent JAK2 inhibitors. It also nicely underscores the relevance of faithful animal models that accurately recapitulate hematological malignancies. Historically these mouse models have focussed on the expression of a single transgene or lesion like Jak2^V617F^. Nevertheless, as techniques like CRISPR/Cas9 become more efficient in introducing specific genetic mutations, the development of new models will continue to help address important clinical questions.

## AUTHOR CONTRIBUTIONS

Charles E. de Bock is the sole author of this article.

## CONFLICT OF INTEREST STATEMENT

The author declares no conflict of interest.

## FUNDING

This research received no funding.

## Data Availability

Data sharing not applicable to this article as no data sets were generated or analyzed during the current study.
